# DachsLife 2015: an investigation of lifestyle associations with the risk of intervertebral disc disease in Dachshunds

**DOI:** 10.1186/s40575-016-0039-8

**Published:** 2016-11-05

**Authors:** R. M. A. Packer, I. J. Seath, D. G. O’Neill, S. De Decker, H. A. Volk

**Affiliations:** 1 Department of Clinical Science and Services, Royal Veterinary College, Hatfield, Hertfordshire UK; 2 Dachshund Breed Council, Flackwell Heath, Buckinghamshire UK; 3 Department of Production and Population Health, Royal Veterinary College, Hatfield, Hertfordshire UK

**Keywords:** Disc disease, Intervertebral, Extrusion, Protrusion, Dachshund, IVDD, Epidemiology

## Abstract

**Background:**

Intervertebral disc disease (IVDD) represents a major problem in the Dachshund, with at a relative risk of IVDD 10–12 times higher than other breeds, and an estimated 19–24 % of Dachshunds showing clinical signs related to IVDD during their lifetime. A variety of genetic, physical and lifestyle-related risk factors for IVDD have previously been identified, with some conflicting findings. As such, advising owners and breeders regarding best-practice for IVDD prevention is challenging at present. This study aimed to (i) estimate prevalence of IVDD in six Dachshund varieties, and (ii) identify risk factors associated with IVDD diagnosis from a wide variety of demographic, conformational, dietary, activity and exercise-related variables.

**Results:**

A web-based survey “Dachs-Life 2015” was carried out from January-April 2015, with responses received for 2031 individual Dachshunds. Three-hundred and ten dogs were classed as Cases based on veterinary-diagnosis of IVDD, and 56 dogs were excluded from further analyses due to a lack of veterinary-diagnosis of their clinical signs. The remaining1665 dogs with no previous signs of IVDD were classified as Non-Cases. The overall prevalence of IVDD was 15.7 % (95 % CI: 14.1–17.3). Breed variety was significantly associated with IVDD risk, with the highest prevalence seen in the Standard Smooth-Haired (24.4 %, 95 % CI: 22.5–26.3) and lowest in the Standard Wire-Haired (7.1 %, 95 % CI: 6.0–8.2). Older dogs and neutered dogs were at increased odds of IVDD. Of the lifestyle risk factors, univariable analysis identified dogs that exercised for <30 min per day, were not allowed to jump on and off furniture, or were supplemented with glucosamine or chondroitin were at increased odds of IVDD, whereas dogs that exercised for more than 1 h per day, that were considered highly or moderately active by their owners, and those that showed at Open or Championship shows were at decreased odds of IVDD.

**Conclusions:**

In line with previous reports, IVDD is commonly diagnosed in the Dachshund, with significant differences in prevalence between Dachshund varieties. Lifestyle risk factors were identified which are hypothesis-generating for future prospective studies, and can inform an evidence-based approach to mitigating IVDD risk for Dachshund owners and breeders.

**Electronic supplementary material:**

The online version of this article (doi:10.1186/s40575-016-0039-8) contains supplementary material, which is available to authorized users.

## Plain English Summary

Intervertebral disc disease (IVDD) is a neurological disorder where the intervertebral discs (the cushions between the veterbrae that make up the spinal column) become diseased and compress the spinal cord, leading to pain, weakness and in some cases, paralysis. IVDD affects many breeds, but is a major problem in the Dachshund breeds in particular. Dachshunds are at a 10–12 times higher risk of IVDD than other breeds, and an estimated 19–24 % of Dachshunds show signs of IVDD during their lifetime.

A variety of genetic, physical and lifestyle-related factors that increase or decrease the risk of IVDD have previously been identified, with some conflicting findings. As such, advising owners and breeders regarding best-practice for IVDD prevention is challenging at present. This study aimed to (i) estimate how common IVDD is in the six Dachshund varieties, and (ii) identify risk factors associated with IVDD diagnosis from a wide variety of demographic, conformational, dietary, activity and exercise-related variables.

A web-based survey “Dachs-Life 2015” was carried out from January-April 2015, with responses received for 2031 Dachshunds. Three-hundred and ten dogs were affected by IVDD. The overall prevalence of IVDD was 15.7 %. The risk of IVDD differed between Dachshund variety, with the highest prevalence seen in the Standard Smooth-Haired (24.4 %) and lowest in the Standard Wire-Haired (7.1 %). Older dogs and neutered dogs were more likely to have IVDD. Of the lifestyle risk factors, dogs that exercised for <30 min per day, were not allowed to jump on and off furniture, or were supplemented with glucosamine or chondroitin were more likely to have IVDD, whereas dogs that exercised for more than 1 h per day, that were considered highly or moderately active by their owners, and those that showed at Open or Championship shows were less likely to have IVDD.

This study helps us to understand how IVDD differs between the Dachshund varieties, and has identified lifestyle risk factors that can be used to advise Dachshund owners how to reduce the risk of IVDD in their dog, advise breeders regarding the breed-associated risks of IVDD, and has identified new avenues for further study of IVDD.

## Background

Intervertebral disc disease (IVDD) is the most common spinal disorder in domestic dogs [1]. IVDD represents a major problem in Dachshunds particularly, with the breed at a relative risk of IVDD 10–12 times higher than other breeds [2, 3], and 19–24 % of Dachshunds are estimated to show clinical signs related to IVDD during their lifetime [3–6]. Histological evidence of intervertebral disc mineralisations, which can result in IVDD, have been reported to be present in 46–48 % of Dachshund intervertebral discs [5, 7]. Increased risk of IVDD in Dachshunds has been primarily attributed to their chondrodystrophic ‘long and low’ conformation [8, 9], with exaggeration of these length-to-height proportions associated with increased risk of disc extrusions [10]. Chondrodystrophy is associated with the expression of a retrogene encoding *fibroblast growth factor 4 (FGF4)* located on chromosome 18 [11]; however, a continuous spectrum of disc degeneration and IVDE/IVDP is seen both among and within chondrodystrophic breeds, suggesting a multi-factorial aetiology involving cumulative effects of several genes and environmental interactions [10]. The Dachshund’s predisposition to IVDD has been demonstrated to be highly heritable [12, 13]; however, reducing the incidence of IVDD is not as simple as devising genetic tests of susceptibility, as has been successfully developed for Lafora’s disease, an autosomal recessive neurological disorder in Miniature Wirehaired Dachshunds [14, 15]. In contrast, IVDD is likely a polygenic disorder with many environmental influences. The pattern of IVDD incidence spans a variety of other breeds, there is wide variety in age at onset of clinical signs, and a number of environmental risk factors have been associated with IVDD, thus indicating a complex, multifactorial aetiology [10, 13, 16–18].

### Risk factors for IVDD

#### Exercise

A variety of environmental and physical risk factors not yet related to an identified disease-related gene mutation have been associated with IVDD, including lifestyle and conformational risk factors. Disc extrusions are most frequently observed at high-motion sites in the vertebral column, such as the thoracolumbar junction, which may indicate that biomechanics have an influence on IVDD [1, 5]. As such, identification of exercise-related risk factors would enable the provision of evidence-based advice on which activities should be promoted or avoided to reduce IVDD risk. To date, little is known about the risk of exercise on IVDD risk, with studies focusing on disc mineralisation rather than disc extrusion [19].

#### Conformation

Conformation and body condition (excess bodyweight) have been implicated as risk factors for IVDD. Increased back length relative to height at the withers (BLHW) has previously been associated with an increased risk of disc extrusions [10]. In addition, Dachshunds with relatively longer backs showed the most severe clinical signs when affected by thoracolumbar IVDE [17]. Conversely, in a study of disc extrusions and protrusions, affected dogs were taller at the withers (as an absolute measure) and had a significantly shorter T1-S1 distance [17]. In contrast, no effect of conformation could be found in a study of clinically confirmed or suspected disc extrusions and disc protrusions [17]. In that study, body condition score (BCS) was not shown to affect the risk of disc extrusions or protrusions [17]; however, increased BCS has been associated with an increased disc extrusion risk [10]. Although not yet investigated, diet may have an effect upon IVDD either via increasing bodyweight (in terms of calorie consumption), or as an independent effect upon disc degeneration (in terms of nutritional value) and requires further investigation. Miniaturisation (smaller body size) has been associated with disc extrusions, which is of particular relevance to the three Miniature Dachshund varieties (Smooth, Wire and Long Haired) [10]. Finally, larger pelvic circumferences have been recorded in IVDD-affected dogs, and shorter tuber calcaneus-to-patellar tendon distance were recorded in affected than in unaffected dogs [17].

#### Breed variety

There are differences in prevalence reports for IVDD between the six Dachshund varieties registered in the UK. Indeed in a previous breed health survey of UK Dachshunds, the average IVDD prevalence was 6.8 % across all varieties; however, this prevalence was elevated in the Standard Smooth-Haired Dachshund at 15.3 % [20]. When stratified by age, prevalence was higher in the older age groups, with 25.5 % of Standard Smooth-Haired Dachshunds affected in the 5–9 years group, and 35.3 % in the 10+ years group, compared to only 4.1 % in the 0–4 years group. It is possible that differences between breed varieties could be attributed to genetic differences, as in the UK each of the six varieties have distinct breeding populations, with potential variation in as yet unidentified IVDD-associated genes. Such variation is also likely within each variety, with markedly higher incidences of IVDD seen in some lines, with an IVDD prevalence as high as 62 % in some Dachshund families [4].

### Aims

There is clearly still much to understand regarding the aetiology and risk factors for IVDD in the Dachshund. This study aimed to report and compare the prevalence of IVDD diagnosed by veterinary surgeons in six Dachshund varieties; the Miniature Wire-Haired (MWH), Miniature Smooth-Haired (MSH), Miniature Long-Haired (MLH), Standard Wire-Haired (SWH), Standard Smooth-Haired (SSH) and Standard Long-Haired (SLH). Additionally, the study aimed to identify risk factors associated with IVDD diagnosis in the six Dachshund varieties across a wide variety of domains: demographic, conformational, dietary, activity and exercise-related. These results can inform an evidence-based approach to advising Dachshund owners on activities to avoid or promote in their dogs to mitigate the risk of IVDD, be hypothesis-generating for future prospective studies of IVDD risk, and advise breeders regarding the breed-associated risks of IVDD.

## Materials and Methods

A web-based survey “Dachs-Life 2015: The UK Dachshund Breed Council’s Back Disease (IVDD) and Lifestyle Survey” was carried out for ten weeks from January 22, 2015 until April 3, 2015 to investigate the prevalence and risk factors for IVDD in Dachshunds. The survey was hosted by the UK Dachshund Breed Council, and owners of Dachshunds with or without a history of IVDD were recruited online via social media (e.g., Facebook) and the UK Dachshund Breed Council’s newsletter. The only exclusion criterion applied to dogs that had already died so that only dogs alive at the time of the survey were included. The survey was primarily aimed at UK-based Dachshund owners, but international owners were also invited to contribute, particularly if their Dachshund had been bred in the UK. Owners of multiple Dachshunds were asked to complete a separate survey for each dog. All owners had the option to remain anonymous throughout the survey.

Owner consent was gained via a statement at the start of the survey stating that they consented for their data to be used for research with the Royal Veterinary College. Personal information was held in accordance with the Data Protection Act 1998, and this project was approved by the RVC Ethics and Welfare Committee (approval number URN 20161602b).

The survey was split into seven sub-sections, (i) About your Dachshund, (ii) Intervertebral Disc Disease, (iii) Exercise, (iv) Activities and Environment, (v) Diet, (vi) General Health and (vii) Owner details.

**Fig. 1:**
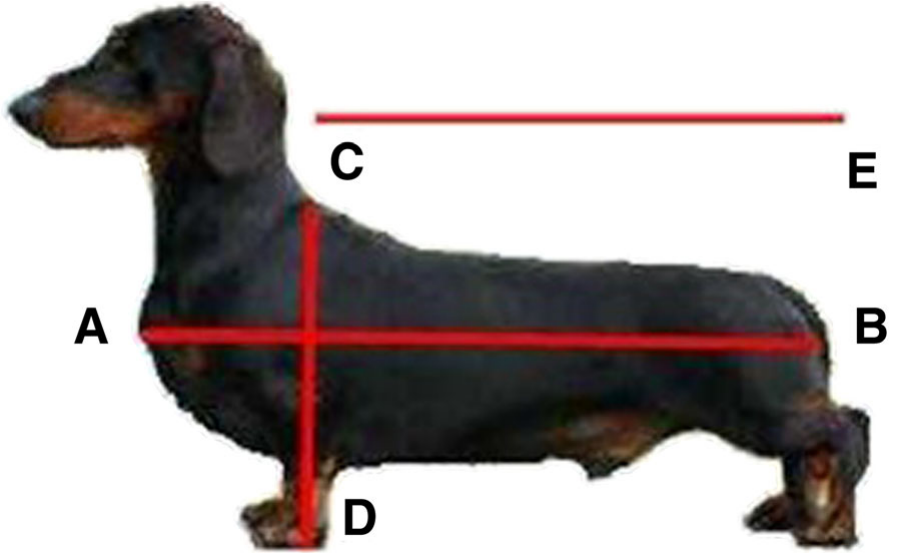
Diagram provided to owners to aid in the collection of conformational measurements. Three conformational measures were measured and reported by the owner: (1) Body length: from point of forechest (**a**) to bottom (**b**), (2) Back length: from withers (**c**) to bottom (**e**), and (3) Height at the withers: from withers (**c**) to ground at the foreleg (**d**). All measures were reported in centimetres

Section (i) About your Dachshund: owners were requested to report their dog’s breed variety, date of birth, sex, neuter status, age at neutering, breeding history (number of litters) and bodyweight (kg). Three conformational measures were measured and reported by the owner: body length from point of forechest (A) to bottom (B) (cm), back length from withers (C) to bottom (B) (cm), and height from withers (C) to ground at the foreleg (D). Owners were provided with a diagram to aid the accurate collection of these measurements (Figure 1). Finally, owners were asked to BCS their dog on a scale of 1–5, from 1 (underweight) to 5 (obese), with BCS 3 classed as ideal. Descriptors and diagrams for each point from 1–5 were provided to aid owner assessment.

Section (ii) Intervertebral Disc Disease: this was an optional section dependent upon whether their dog had ever displayed any signs of IVDD (which were described as “IVDD (back problems)” throughout) or not. Owners were asked an initial filter question of whether their dog had any history of IVDD (back problems), with those who answered “No” directed to section (iii) and used as Non-Cases for risk factor analyses. Owners who answered “Don’t know”, were directed to section (iii), and were excluded from risk factor analyses due to the uncertainty of their IVDD status. For owners who stated “Yes”, further questions were asked regarding their diagnosis and treatment, to determine whether they could be classed as Cases in the risk factor analyses. To assess the certainty of IVDD diagnosis, owners were asked who diagnosed their dog with IVDD: (a) No veterinary diagnosis; presumed by owner based on clinical signs dog was showing; or (b) Diagnosed by a veterinary surgeon (. Only those dogs diagnosed by a veterinary surgeon were classed as Cases, due to the inherent uncertainty of an owner-based diagnosis. As such, group (a) dogs were excluded from further risk factor analyses. Owners of affected dogs were also asked to report the location of the problem disc(s) as cervical or thoracic (or both), diagnostic tests carried out, the severity of clinical signs associated with IVDD (from pain and discomfort through to paralysis), whether they were treated with cage rest, medication and/or surgery, whether there was subsequent IVDD episodes, age at first episode, age at subsequent episodes, onset of clinical signs (from <1 day to >1 month), and whether there was a family history of IVDD (and which relative).

Section (iii) Exercise: requested owners to report their dog’s current exercise routine and activities, and exercise during their growth/development. Owners reported how much exercise their Dachshund undertook as a puppy (under 12 months old) as a multiple of their age in months: less than 5 min per month of age, 5 min per month of age or more than 5 min per month of age. They then reported how much exercise their dog receives (up to 30 min, 30 min – 1 h, over 1 h) on a typical weekday and on a typical weekend, for different activities: free running/playing in the garden, walking on the lead, mixed walking on and off the lead, and accompanying the owner while jogging/cycling. Owners reported whether their dog was walked on a lead, harness or neither, and if they pulled on their lead or harness. Finally, owners assessed how active they considered their dog: mildly active, moderately active, or highly active.

Section (iv) Activities and Environment: Owners were requested to report specific activities their dog was involved in from the following: Kennel Club Good Citizen Dog Scheme, Cani-X, earth/working trials, heelwork to music, mini agility, obedience, participation in Facebook group walks/activities, showing at fun/charity/exemption shows, showing at Kennel Club Championship shows, showing at Kennel Club Open shows, therapy dog (Pets as Therapy). Owners were asked if their dog had daily company with other dogs (number and Dachshunds/non-Dachshunds), whether they regularly use stairs in their daily routine (a flight of stairs each day, only a step in/out of the house each day, no stair use), and whether their dog regularly jumped on and off furniture in their daily routine (yes/no).

Section (v) Diet: Owners reported the type of diet their dog was fed, aged under and over 12 months of age. Owners selected from complete dry food, wet food, or bones and raw food (BARF), or combinations of these diets. Owners additionally reported whether they gave their Dachshund any dietary supplements or additives from the following: coconut oil, glucosamine, chondroitin, Plaque-off™, Vitamin C or multi-vitamins. Finally, owners reported whether their dog was regularly fed treats in addition to their normal diet from the following: table scraps, other human food (e.g., scrambled egg), dog treats or dog biscuits.

Section (vi) General Health: In addition to reporting their dog’s IVDD history, owners were asked to report if their dog had been diagnosed by their veterinary surgeon with other diseases from the following: adverse reaction to vaccination, arthritis, auto-immune disease, blindness (clinical symptoms, not DNA test), cancers or tumours (except mammary), Cushing’s disease, deafness, distichiasis, epilepsy, heart disease, heart murmur, kidney disease, Lafora disease (clinical symptoms, not DNA test), liver disease, mammary tumour(s), patella luxation or skin allergy. Owners reported if their dog had been vaccinated in the past 12 months using conventional vaccines (i.e., not homeopathic), and whether and how often they receive booster vaccinations.

Section (vii) Owner details: Owners reported the acquisition of their dog with regards to the age of the dog, and provenance from the following: bred this Dachshund themselves, Kennel Club Assured Breeder, Breed Club member, show breeder, hobby breeder (1^st^ litter), hobby breeder (they have bred before or since), commercial breeder (advertises and sells multiple breeds), puppy farm (N.B. may not have realised this at the time of purchase), pet shop, rescue organisation, or imported to the UK. Owners were optionally requested to provide their Dachshunds Kennel Club registered name, and finally, if their Dachshund was bred in the UK, and what country they currently live in.

### Statistical analysis

Before importation into IBM SPSS version 21 statistical software for analysis, data were imported into Microsoft Excel 2010 for cleaning. From the raw conformational measures, back length: height at the withers (BL:HW) and total length: height at the withers (TL:HW) were calculated by dividing back or total body length by height at the withers, respectively. The continuous variables age, BL:HW and TL:HW were categorised by calculating quartiles to identify suitable category limits. The primary outcome measure was IVDD status, coded binomially as Case (1) or Non-Case (0). Dogs whose owners were unsure as to their IVDD status, or those where those had made the diagnosis themselves without a veterinary diagnosis were excluded from analyses. Prevalence values with 95 % confidence intervals (95 % CI) were reported separately for the overall study population of Dachshunds, and for each breed variety. The 95 % CI estimates were derived from standard errors based on approximation to the normal distribution for disorders with ≥10 events [21]. Descriptive statistics characterised the sex/neuter, age, bodyweight, BCS and conformational measures for the six Dachshund varieties under investigation, with the Kruskall-Wallis (KW) test used to compare age and bodyweight between varieties, and the chi-squared test (*X* ^2^) used to compare sex and BCS between varieties. Mean ± SD was reported where the data were normally distributed and otherwise the median (IQR) was reported. Univariable binary logistic regression was used to assess associations between risk factors (signalment, conformational, exercise and activities, diet) and a diagnosis of IVDD. As 50 variables were tested for their association with IVDD, the Bonferroni correction [21] was applied to correct for multiple testing (0.05/50 = 0.001) and *P*-values <0.001 were considered significant. Due to the large number of exercise, activity and diet related variables and their potential interactions, these variables were only described at the univariable level for the purposes of further study and hypothesis generation. Broadly significant signalment and conformational variables from the univariable analysis (*P* ≤0.2) were taken forward for consideration using multivariable binary logistic regression modelling [22]. Collinearity of variables taken forward was explored via standard statistical methods [23]. A manual forward selection step-wise construction method was taken for model building. The forward step-wise regression used the likelihood ratio test. Final variables were evaluated for pairwise interactions and the final model was evaluated with the Hosmer-Lemeshow goodness-of-fit test [24].

## Results

Responses were received from the owners of 2031 Dachshunds, all of which gave consent to use their data for research purposes. The majority of owners were UK-based (83.9 %, *n* = 1704), followed by Australia (9.5 %, *n* = 193), USA (3.4 %, *n* = 69), and the rest of Europe (1.2 %, *n* = 24). Less than 1 % in total were based in Canada, Bermuda, the Channel Islands, Hong Kong, India, Ireland, Japan, New Zealand, South Africa and UAE.

### Demographics

**Fig. 2:**
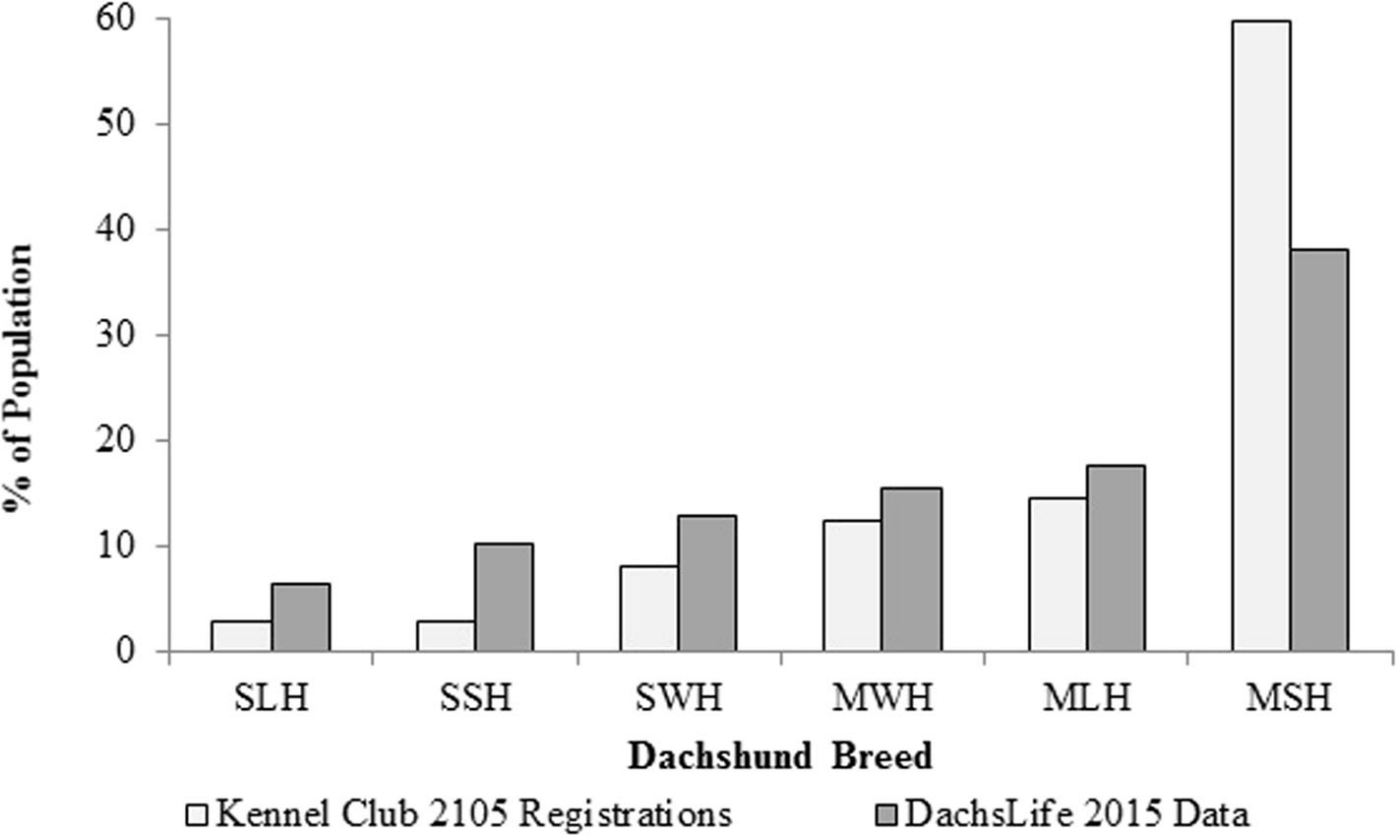
Comparative popularity of the six Dachshund varieties in the DachsLife2015 dataset versus Kennel Club registrations. The popularity of the six Dachshund varieties in the dataset for DachsLife2015 was similar to their proportional registration with the Kennel Club in 2015. The Miniature Smooth Haired (MSH) Dachshund was most represented in both populations. SLH = Standard Long Haired, SSH = Standard Smooth Haired, SHW = Standard Wire Haired, MWH = Miniature Wire Haired, MLH = Miniature Long Haired, MSH = Miniature Smooth Haired

All six varieties of Dachshunds were represented, with the most popular variety the MSH (37.9 %, *n* = 769), followed by the MLH (17.6 %, *n* = 358) and MWH (15.4 %, *n* = 312). The Standard varieties were less represented than the Miniature varieties, with 12.7 % SWH (*n* = 258), 10.2 % SSH (*n* = 207) and 6.3 % SLH (*n* = 127). The popularity of Dachshund varieties in this dataset was similar to their proportional registration with the Kennel Club in 2015 (Figure 2).

The majority of Dachshunds were bred in the UK (83.7 %, *n* = 1689). The origin of the Dachshunds varied, with 9.7 % bred by the owner (*n* = 197), 0.6 % imported (*n* = 13), 4.2 % rescued/rehomed (*n* = 86), 1.0 % from puppy farms (*n* = 21), and 1.0 % bought from pet shops (*n* = 20). The majority of dogs were from breeders, with 48.1 % reported to be from Kennel Club Assured Breeders (*n* = 974, although this category may have been confused with simply Kennel Club registered breeders), 16.2 % from breed club members (*n* = 328), 15.1 % from Show breeders (*n* = 307), 6.5 % from first time hobby breeders (*n* = 132), 13.4 % from hobby breeders who have bred before or since (*n* = 272), and 2.4 % from commercial breeders that advertise and sell multiple breeds (*n* = 49). The most common age of acquisition was 8 weeks (32.5 %), followed by 9–10 weeks (22.3 %), 11–12 weeks (16.2 %) and over 12 weeks (15.6 %). The least common ages of acquisition were under 8 weeks (3.7 %) and puppies who were bred by their owners, and owned from birth (9.7 %).

The median age (months) (IQR) at the time of the study was 50.2 months (24–83.7 months). The youngest dog in the dataset was 2 months old, and the oldest was 231.4 months old. Of the 2031 dogs, 209 (10.3 %) were puppies (under 1 year old). There was an even sex and neuter status distribution, with 23.7 % male entire, 20.0 % female entire, 28.5 % female neutered, and 27.8 % male neutered. Of the neutered animals, the most common age at neutering was 6–12 months (46.1 %, *n* = 529), followed by 1–2 years (17.5 %, *n* = 201) and under 6 months (9.7 %, *n* = 111). The remainder (26.2 %, *n* = 301) were neutered after 2 years of age. The majority of dogs had not had, or sired a litter of puppies (82.6 %), with 7.2 % having had or sired 1 litter, 4.7 % 2 litters, 3.4 % 3 litters, with 2.0 % of owners unsure. Males were less likely to have been used in breeding, with 88.6 % of males having never been used in breeding, compared to 76.4 % of females (*X* ^2^=, *p* < 0.001). Significant differences between Dachshund varieties were found for bodyweight and age (Table 1). The mean bodyweight (kg) ± SD of the three miniature varieties (MSH 5.4, MLH 5.5, MWH 5.5) was lower than those of the standard varieties (SSH 9.5, SLH 12.6, SWH 11.2) (*p* < 0.001) (Table 1). The median age (months) was lowest in the MSH (41.1) compared to the other five varieties, and was highest in the SLH (66.2) (*p* < 0.001) (Table 1). Sex/neuter status varied significantly between varieties, for example, the SWH variety had fewer male neutered dogs (17.1 %) and more male entire dogs (29.5 %) than other varieties (*p* < 0.001). The distribution of body condition scores (1–5) differed significantly between varieties, with fewer MLH dogs at an ideal bodyweight (BCS = 3, 74.2 %) and more in the overweight or obese categories (BCS = 4 and 5; 10.1 %) than the other varieties (*p* = 0.044).

**Table 1:** Demographic comparison between six Dachshund varieties, reported by their owners as part of the DachsLife 2015 survey (*n* = 2031)

Variable	Category	MSH	MLH	MWH	SSH	SLH	SWH	Significant difference?
Age (months)	Median (IQR)	41.1 (18.6–71.8)	62.7 (28.9–95.9)	52.5 (27.2–88.9)	58.8 (29.4–94.5)	66.2 (39.8–92.0)	52.5 (27.5–88.9)	KW: 70.8, *P* < 0.001
Bodyweight (kg)	Median (IQR)	5.4 (4.5–6.5)	5.5 (4.6–6.5)	5.5 (4.8–6.5)	9.5 (6.4–11.5)	12.6 (10.0–15.0)	11.2 (10.0–12.7)	KW: 853.6, *P* < 0.001
Sex/neuter	Female entire % (n)	19.0 % (146)	19.6 % (70)	21.5 % (67)	14.5 % (30)	18.9 % (24)	26.7 % (69)	*X* ^2^ = 41.9, *P* < 0.001
	Female neutered % (n)	29.4 % (226)	28.5 % (102)	30.4 % (95)	23.7 % (49)	29.9 % (38)	26.7 % (69)	
	Male entire % (n)	22.2 % (171)	20.1 % (72)	25.3 % (79)	25.1 % (52)	25.2 % (32)	29.5 % (76)	
	Male neutered % (n)	29.4 % (226)	31.8 % (114)	22.8 % (71)	36.7 % (76)	26.0 % (33)	17.1 % (44)	
Body condition score	1 % (n)	2.3 % (18)	3.9 % (14)	3.9 % (12)	2.9 % (6)	0.8 % (1)	3.5 % (9)	*X* ^2^ = 31.9, *P* = 0.044
	2 % (n)	8.5 % (65)	11.8 % (42)	8.1 % (25)	11.2 % (23)	5.6 % (7)	7.4 % (19)	
	3 % (n)	82.4 % (631)	74.2 % (264)	78.5 % (241)	81.6 % (168)	85.7 % (108)	84.8 % (218)	
	4 % (n)	6.4 % (49)	9.3 % (33)	9.1 % (28)	3.9 % (8)	6.3 % (8)	4.3 % (11)	
	5 % (n)	0.4 % (3)	0.8 % (3)	0.3 % (1)	0.5 % (1)	1.6 % (2)	0.0 % (0)	

### Conformation

Significant differences between Dachshund varieties were found for all three conformational measures (BL, LH, HW), and both ratios of body/back length to height (Table 2). The variety with the longest BL:HW ratio was the MLH, followed by the MSH, with the SWH having the shortest. The varieties with the longest TL:HW were also the MLH, followed by the MSH, with the SWH again the shortest.

**Table 2:** Conformational comparison between six Dachshund varieties, measured and reported by their owners as part of the DachsLife 2015 survey (*n* = 2031)

Breed variety	Back length (BL) (cm)	Total length (LH) (cm)	Height at the withers (HW) (cm)	BL:HW	TL:HW
MSH	34.80 (5.25)	40.58 (5.80)	20.27 (3.58)	1.75 (0.31)	2.04 (0.35)
MLH	36.23 (5.24)	42.44 (5.93)	20.85 (3.66)	1.76 (0.26)	2.06 (0.29)
MWH	35.15 (4.94)	40.51 (5.38)	20.83 (3.12)	1.71 (0.26)	1.96 (0.29)
SSH	41.24 (5.61)	48.57 (7.45)	24.72 (4.89)	1.72 (0.33)	2.01 (0.37)
SLH	47.93 (8.31)	55.86 (8.85)	27.77 (4.78)	1.74 (0.24)	2.03 (0.25)
SWH	45.26 (6.06)	52.82 (5.95)	27.00 (3.60)	1.69 (0.26)	1.98 (0.26)
Significant difference?	F = 236.60, *P* < 0.001	F = 269.41, *P* < 0.001	F = 201.62, *P* < 0.001	F = 2.62, *P* = 0.23	F = 4.39, *P* < 0.001

Figures represent mean values (SD). *BLHW* Back length: height at the withers, *TLHW* Total length: height at the withers

#### Exercise

The most common responses on the amount of exercise their dog received as a puppy (<12 months old) were ‘more than 5 min per month of age’ (44.6 %, *n* = 895), followed by ‘5 min per month of age’ (17.1 %, *n* = 343). A minority of owners exercised their puppy for ‘less than 5 min per month of age’ (9.5 %, n-193), and 16.9 % (*n* = 343) of owners could not remember/didn’t know. Owner’s self-reports of their dog’s activity were primarily ‘moderately active’ (48.8 %, *n* = 991), followed by ‘highly active’ (28.3 %, *n* = 575), ‘mildly active’ (20.0 %, *n* = 405), and ‘not at all active’ (2.9 %, *n* = 58). The amount of time spent active on weekdays (a combination of walks and free running/playing in the garden) varied between dogs, from less than 30 min per day (14.2 %, *n* = 284), 30 min to 1 h (28.4 %, *n* = 567) to the most common; over 1 h per day (56.9 %, *n* = 1136). While on walks, 38.2 % (*n* = 762) of dogs were allowed off lead time. A minority of dogs accompanied their owners while jogging or cycling (0.9 %, *n* = 18). The majority of dogs were walked on a collar and lead (64.2 %, *n* = 1286), followed by use of a harness (*n* = 28.7 %, *n* = 575). A minority of owners used a combination of collar and harness (0.5 %, *n* = 10) or never used either (6.5 %, *n* = 131). Of the dogs walked on a collar, 24.7 % were reported to pull (*n* = 320), and of the dogs that walked on a harness, 36.0 % were reported to pull (*n* = 211).

#### Activities and environment

The most popular activity that Dachshunds participated in beyond walking was obedience training (21.4 %) (Additional file 1: Table S1). The most common place Dachshunds spent their day was in the house, with no restriction on which rooms (47.6 %, *n* = 967), followed by in the house but restricted to certain rooms (46.1 %, *n* = 937). A small minority of dogs spent most of their day outdoors/in the garden (2.3 %, *n* = 46), in a dog cage (1.7 %, *n* = 34), in an outdoor kennel and run (1.2 %, *n* = 34), in a play pen (0.5 %, *n* = 11), in an outbuilding with run (0.4 %, *n* = 8), or in an outbuilding without a run (0.1 %, *n* = 3). While in the house, over two thirds of dogs were allowed to regularly jump on and off furniture (67.4 %, *n* = 1368). Around one third of dogs did not use stairs each day (34.9 %, *n* = 708), and conversely 37.0 % (*n* = 751) used a flight of stairs every day, while some dogs only used an up/down step to enter and exit their house each day (28.20 %, *n* = 572). The most common canine company for Dachshunds was none, with 34.7 % of Dachshunds being an only dog (*n* = 705), while 24.2 % (*n* = 491) had the daily company of one other Dachshund, and 18.7 % (*n* = 380) the company of 2 or more other Dachshunds. A smaller proportion of Dachshunds had the company of one other dog who is not a Dachshund (10.3 %, *n* = 209), or several other dogs including non-Dachshunds (12.1 %, *n* = 246).

#### Diet

Owners fed their Dachshunds either a complete dry, wet or raw diet, or a combination of these diets. The most common diet was a complete dry diet (40.6 %, Additional file 1: Table S2) followed by a combination of dry complete and wet diets (24.8 %). Raw food (BARF) diet alone (7.8 %) or in combination with complete and wet (6.5 %) or wet (1.9 %) were the least common dietary options. In addition to their main diet, 91.8 % of dogs (*n* = 1864) also received treats. The most common type of treats fed were dog treats (53.8 %) and dog biscuits (37.8 %) (Table 6). Around one third of dogs received supplements in addition to their main diet (33.7 %, *n* = 666), the most common being coconut oil (8.5 %).

#### General health

The most prevalent diagnoses other than IVDD in this population of Dachshunds was skin allergies (13.1 %), followed by heart murmurs (3.5 %) and arthritis (3.1 %) (Additional file 1: Table S3). The majority of Dachshunds had been vaccinated (91.3 %, *n* = 1838), and the majority of dogs received annual boosters (74.2 %).

### IVDD

Of the 2031 dogs in the study, 310 dogs had a IVDD diagnosis from their veterinary surgeon (*n* = 113 from their first opinion veterinary surgeon, *n* = 197 from their first opinion veterinary surgeon and referral to a neurologist) (Additional file 2). These dogs were classed as Cases. Twenty-five owners were unsure about whether their dog had experienced signs of IVDD previously, and 31 owners presumed their dog had experienced IVDD based on their clinical signs but had not been diagnosed by a veterinary surgeon. These dogs (*n* = 56) were excluded from further analyses due to the uncertainty of their diagnosis. The remaining dogs (*n* = 1665) with no previous signs of IVDD were classified as Non-Cases.

#### Prevalence

The overall prevalence of IVDD was 15.7 % (95 % CI: 14.1–17.3), with the highest prevalence seen in the SSH (24.4 %, 95 % CI: 22.5–26.3) and lowest in the SWH (7.1 %, 95 % CI: 6.0–8.2). Compared to the SWH, four varieties were at a significantly increased odds of IVDD (MSH, MLH, MWH, SSH), with the SSH at a 4.19 increased odds of IVDD (*p* < 0.001) (Table 3).

**Table 3:** Prevalence and age of onset of IVDD in six varieties of Dachshunds and results of a univariable logistic regression (OR = odds ratio, CI = confidence interval)

Breed	Cases (n)	Total (n)	Age at IVDD onset (median years, IQR)	IVDD prevalence (%)	95 % CI (%)	OR	95 % CI (OR)	*p*
Standard Wire-Haired	18	252	7.0 (5.0–8.3)	7.1	5.97–8.23	1 (base)	–	<0.001
Standard Smooth-Haired	49	201	6.0 (4.0–7.0)	24.4	22.51–26.29	4.19	2.35–7.47	<0.001
Miniature Wire-Haired	54	305	5.0 (4.0–6.0)	17.7	16.02–19.38	2.80	1.59–4.91	<0.001
Miniature Smooth-Haired	127	744	5.0 (4.0–6.0)	17.1	15.44–18.76	2.68	1.60–4.48	<0.001
Miniature Long-Haired	46	346	6.0 (5.0–7.5)	13.3	11.80–14.8	1.99	1.13–3.53	0.018
Standard Long-Haired	16	127	6.0 (3.0–6.8)	12.6	11.14–14.06	1.87	0.92–3.81	0.083

#### Diagnosis and treatment

Of the 310 veterinarian-diagnosed cases, the diagnostic processes included MRI *n* = 121 (39.0 %), plain radiography *n* = 118 (38.1 %), myelography *n* = 43 (13.9 %) and CT *n* = 41 (13.2 %). Based on clinical signs, the causal disc was thought to be in the thoracolumbar region in 88.1 % of cases (*n* = 273), with a minority thought to be in the cervical region (7.7 %, *n* = 24) or unknown to the owner (4.2 %, *n* = 13). The mean ± SD age at diagnosis was 5.42 years ± 2.40. The onset of IVDD related clinical signs was most commonly less than 24 h (41.7 %, *n* = 128), followed by 1–3 days (32.6 %, *n* = 100) and 4–7 days (8.8 %, *n* = 27). A minority of cases had a more chronic onset of more than 1 week (8.1 %, *n* = 25) or more than 1 month (7.2 %, *n* = 22). Severity of clinical signs varied between dogs, with 17 dogs (5.6 %) presenting with pain and discomfort but no neurological deficits. One quarter of cases (*n* = 25.2 %, *n* = 77) were ataxic upon presentation but able to walk; however, the majority of cases (69.3 %, *n* = 212) were unable to walk upon presentation. Sixty percent (60.1 %, *n* = 184) of cases were treated surgically, with the remaining 39.9 % (*n* = 122) treated with cage rest and medication only. Of dogs for which radiographs were their only diagnostic imaging modality (*n* = 65), the majority were only treated with cage rest and medication only (*n* = 52, 80.0 %, *X* ^2^ = 104.5, *p* < 0.001). In contrast, of dogs that had advanced diagnostic imaging (CT and/or MRI, *n* = 148), only 16.0 % (*n* = 12) had been treated with cage rest and medication only (*X* ^2^ = 81.6, *p* < 0.001). A known family history of IVDD was reported in *n* = 25 cases (8.06 %), *n* = 8 dams, *n* = 12 sires, *n* = 21 siblings and *n* = 4 offspring.

### Risk factors for IVDD

#### Demographic and conformational risk factors

Univariable analysis identified three of the eight demographic and conformational risk factors were associated with IVDD risk (*p* < 0.001, Table 6, Table 7). The proportion of each Dachshund variety in case and non-case groups significantly differed, with SSH, MWH and MSH more frequently represented as cases when compared to the SWH (Table 6). Increasing age was associated with increased odds of IVDD as both a continuous variable and when categorised, with dogs aged 8–10 years old at the highest odds of being affected by IVDD compared to 0–2 years. Entire dogs were at significantly decreased odds of IVDD. Sex, bodyweight, BCS and conformational variables (BLHW, TLHW) were not associated with IVDD risk (>0.001) (Table 4).

**Table 4:** Risk factors (signalment and physical factors) for intervertebral disc disease (IVDD) from univariable analysis (SD = standard deviation, OR = odds ratio, CI = confidence interval)

Variable	Category	Case	Non-Case	OR	95 % CI	*p*
Age (continuous)	Mean ± SD	96.50 ± 33.89	50.6 ± 38.1	1.03	1.02–1.03	<0.001
Age (categorical)	0–2 years	1 (0.2 %)	497 (99.8 %)	1 (base)		
	2–4 years	18 (3.9 %)	443 (96.1 %)	20.19	2.69–151.89	0.004
	4–6 years	50 (13.6 %)	318 (86.4 %)	78.15	10.74–568.51	<0.001
	6–8 years	99 (33.0 %)	201 (67.0 %)	244.79	33.91–1767.01	<0.001
	8–10 years	75 (41.9 %)	104 (58.1 %)	358.41	49.28–2607.02	<0.001
	>10 years	67 (39.6 %)	102 (60.4 %)	326.46	44.81–2378.69	<0.001
Neuter status	Neutered	231 (20.9 %)	874 (79.1 %)	1 (base)		
	Entire	79 (9.1 %)	791 (90.9 %)	0.38	0.29–0.50	<0.001
Sex	Male	154 (15.2 %)	861 (84.8 %)	1 (base)		
	Female	156 (16.3 %)	804 (83.8 %)	1.09	0.85–1.38	0.511
Bodyweight (continuous)	Mean ± SD	7.14 ± 3.23	7.32 ± 3.47	0.98	0.95–1.02	0.399
BCS	5	1 (10.0 %)	9 (90.0 %)	1 (base)		0.681
	4	26 (19.7 %)	106 (80.3 %)	2.21	0.27–18.21	0.462
	3	244 (15.4 %)	1341 (84.6 %)	1.64	0.21–12.98	0.641
	2	28 (15.9 %)	148 (84.1 %)	1.70	0.21–13.98	0.620
	1	11 (18.6 %)	48 (81.4 %)	2.06	0.24–18.02	0.513
BLHW (categories)	<1.5	95 (14.2 %)	575 (85.8 %)	1 (base)		0.744
	1.5–1.7	76 (15.6 %)	412 (84.4 %)	1.12	0.81–1.55	0.509
	1.7–1.9	49 (16.8 %)	242 (83.2 %)	1.23	0.84–1.79	0.289
	>1.9	59 (15.6 %)	319 (84.4 %)	1.12	0.79–1.59	0.530
TLHW (categories)	<1.8	73 (17.9 %)	334 (82.1 %)	1 (base)		0.223
	1.8–2.0	91 (13.3 %)	594 (86.7 %)	0.70	0.50–0.98	0.038
	2.0–2.2	58 (15.7 %)	312 (84.3 %)	0.85	0.58–1.24	0.401
	>2.2	57 (15.6 %)	308 (84.4 %)	0.85	0.58–1.24	0.390

#### Exercise, activities and environment

Univariable analysis identified six of the 23 exercise and activity related variables were associated with IVDD risk (*p* < 0.001, Table 5). Dogs that exercised for less than 30 min per day were at an increased odds of having IVDD, whereas dogs that exercised for more than 1 h per day were at a reduced odds of IVDD. Dogs that were considered to be highly active or moderately active were at a reduced odds of IVDD compared to dogs considered not at all active. Dogs who were not allowed to jump on and off furniture were at an increased odds of IVDD compared to those who were allowed. Dogs that were involved in showing at either Championship shows or Open shows were at a reduced odds of being affected by IVDD than those that did not (Table 5).

**Table 5:** Risk factors (exercise, environment and activity factors) for intervertebral disc disease (IVDD) from univariable analysis (OR = odds ratio, CI = confidence interval)

Variable	Category	Case	Non-Case	OR	95 % CI	*p*
Less than 30 min exercise	No	231 (13.8 %)	1440 (86.2 %)	1 (base)		
	Yes	77 (28.5 %)	193 (71.5 %)	2.49	1.85–3.52	<0.001
30 min–1 h exercise	No	212 (15.3 %)	1176 (84.7 %)	1 (base)		
	Yes	96 (17.4 %)	457 (82.6 %)	1.17	0.90–1.52	0.257
Over 1 h exercise	No	176 (21.2 %)	654 (78.8 %)	1 (base)		
	Yes	132 (11.9 %)	979 (88.1 %)	0.50	0.39–0.64	<0.001
Off lead exercise	No	204 (17.1 %)	992 (82.9 %)	1 (base)		
	Yes	103 (13.9 %)	640 (96.1 %)	0.78	0.61–1.01	0.061
Jogging/cycling with owner	No	307 (16.0 %)	1616 (84.0 %)	1 (base)		
	Yes	1 (5.9 %)	16 (94.1 %)	0.33	0.04–2.49	0.282
Collar use	Neither	23 (18.4 %)	102 (81.6 %)	1 (base)		0.016
	Both	1 (10.0 %)	9 (90.0 %)	0.49	0.06–4.08	0.512
	Collar	172 (13.7 %)	1086 (86.3 %)	0.70	0.44–1.14	0.149
	Harness	107 (19.3 %)	447 (80.7 %)	1.06	0.64–1.75	0.815
Pulling	No pull	217 (15.8 %)	1159 (84.2 %)	1 (base)		
	Harness pull	35 (17.6 %)	164 (82.4 %)	1.14	0.77–1.69	0.514
	Collar pull	39 (12.5 %)	273 (87.5 %)	0.76	0.53–1.10	0.147
Obedience	No	237 (15.5 %)	1290 (84.5 %)	1 (base)		
	Yes	63 (15.1 %)	353 (84.9 %)	0.97	0.72–1.31	0.851
Participation in group walks	No	260 (15.9 %)	1378 (84.1 %)	1 (base)		
	Yes	40 (13.1 %)	265 (86.9 %)	0.80	0.56–1.14	0.222
Showing at fun/charity/exemption shows	No	276 (16.1 %)	1434 (83.9 %)	1 (base)		
	Yes	24 (10.3 %)	209 (89.7 %)	0.59	0.38–0.93	0.022
Showing at Kennel Club Championship shows	No	291 (16.9 %)	1431 (83.1 %)	1 (base)		
	Yes	9 (4.1 %)	212 (95.9 %)	0.21	0.11–0.41	<0.001
Showing at Kennel Club Open shows	No	289 (16.7 %)	1441 (83.3 %)	1 (base)		
	Yes	11 (5.2 %)	202 (94.8 %)	0.27	0.15–0.51	<0.001
Canine Good Citizen	No	286 (16.1 %)	1485 (83.9 %)	1 (base)		
	Yes	14 (8.1 %)	158 (91.9 %)	0.46	0.26–0.81	0.007
Mini agility	No	288 (15.5 %)	1574 (84.5 %)	1 (base)		
	Yes	12 (14.8 %)	69 (85.2 %)	0.87	0.51–1.78	0.874
Earth/Working trials	No	292 (15.5 %)	1596 (84.5 %)	1 (base)		
	Yes	8 (14.5 %)	47 (85.5 %)	0.93	0.44–1.99	0.852
Therapy dog (Pets as Therapy)	No	295 (15.5 %)	1609 (84.5 %)	1 (base)		
	Yes	5 (12.8 %)	34 (87.2 %)	0.80	0.31–2.07	0.648
Heelwork to music	No	299 (15.4 %)	1638 (84.6 %)	1 (base)		
	Yes	1 (16.7 %)	5 (83.3 %)	1.10	0.13–9.41	0.934
Cani-X	No	298 (15.4 %)	1640 (84.6 %)	1 (base)		
	Yes	2 (40.0 %)	3 (60.0 %)	0.16	0.61–22.05	0.155
Spend day	Dog play pen	1 (10.0 %)	9 (90.0 %)	1 (base)		0.724
	House – no restriction	152 (16.1 %)	791 (83.9 %)	1.73	0.22–13.75	0.605
	House – restrictions	144 (15.9 %)	764 (84.1 %)	1.70	0.21–13.49	0.617
	Outbuilding with run	0 (0 %)	8 (100.0 %)	0.00	–	0.999
	Outdoor/farm/garden	4 (9.1 %)	40 (90.9 %)	0.90	0.09–9.05	0.929
	Kennel and run	0 (0 %)	24 (100.0 %)	0.00	–	0.998
	Outbuilding no run	0 (0 %)	3 (100 %)	0.00	–	0.999
	Dog cage/crate	9 (26.5 %)	25 (73.5 %)	3.24	0.36–29.30	0.295
Other dog company	Only dog	112 (16.4 %)	573 (83.6 %)	1 (base)		0.350
	One other Dachshund	85 (17.9 %)	391 (82.1 %)	1.11	0.82–1.52	0.501
	One non-Dachshund	25 (12.3 %)	178 (87.7 %)	0.72	0.45–1.14	0.164
	2 or more Dachshunds	54 (14.6 %)	315 (85.4 %)	0.88	0.62–1.25	0.466
	2 or more non Dachshunds	34 (14.0 %)	208 (86.0 %)	0.84	0.55–1.23	0.399
Stairs	Up and down flight of stairs each day	90 (12.3 %)	641 (87.7 %)	1 (base)		0.005
	No stairs each day	116 (17.0 %)	567 (83.0 %)	1.46	1.10–1.96	0.013
	One step in/out of house each day	104 (18.5 %)	457 (81.5 %)	1.62	1.19–2.20	0.002
Furniture jump	Yes	138 (10.4 %)	1194 (89.6 %)	1 (base)		
	No	172 (26.7 %)	471 (73.3 %)	3.16	2.47–4.05	<0.001
Activity level	Not at all active	20 (37.7 %)	33 (62.3 %)	1 (base)		<0.001
	Highly active	44 (7.8 %)	517 (92.2 %)	0.140	0.07–0.27	<0.001
	Moderately active	133 (13.8 %)	832 (86.2 %)	0.264	1.45–0.47	<0.001
	Mildly active	113 (28.6 %)	282 (71.4 %)	0.661	0.36–1.20	0.174

#### Diet

Univariable analysis identified two of the 18 diet, treat and supplement related variables were associated with IVDD risk (*p* < 0.001, Table 6). Dogs supplemented with glucosamine or chondroitin were at an increased odds of IVDD than those that were not (Table 6). No significant associations with diet or treats were found.

**Table 6:** Risk factors (diet-based factors) for intervertebral disc disease (IVDD) from univariable analysis (OR = odds ratio, CI = confidence interval)

Variable	Category	Case	Non-Case	OR	95 % CI	*p*
Treats- Table scraps	No	241 (15.2 %)	1346 (84.8 %)	1 (base)		
	Yes	69 (17.8 %)	319 (82.2 %)	1.21	0.90–1.62	0.208
Treats- Other human food	No	249 (15.1 %)	1400 (84.9 %)	1 (base)		
	Yes	61 (18.7 %)	265 (81.3 %)	1.29	0.95–1.76	0.102
Treats- Dog treats	No	137 (15.1 %)	772 (84.9 %)	1 (base)		
	Yes	173 (16.2 %)	893 (83.8 %)	1.09	0.86–1.39	0.481
Treats- Biscuits	No	206 (16.8 %)	1021 (83.2 %)	1 (base)		
	Yes	104 (13.9 %)	644 (86.1 %)	0.80	0.62–1.03	0.088
Treats- Other treats	No	236 (15.5 %)	1290 (84.5 %)	1 (base)		
	Yes	74 (16.5 %)	375 (83.5 %)	1.08	0.81–1.44	0.603
Diet – Raw (only)	No	278 (17.2 %)	1336 (82.8 %)	1 (base)		
	Yes	25 (18.1 %)	113 (81.9 %)	0.94	0.60–1.48	0.790
Diet – Complete Dry (only)	No	177 (17.1 %)	860 (82.9 %)	1 (base)		
	Yes	126 (17.6 %)	589 (82.4 %)	0.962	0.75–1.24	0.763
Diet – Wet (only)	No	271 (17.0 %)	1320 (83.0 %)	1 (base)		
	Yes	32 (19.9 %)	129 (80.1 %)	0.83	0.55–1.25	0.364
Diet – Raw + Complete Dry	No	278 (17.4 %)	1320 (82.6 %)	1 (base)		
	Yes	25 (16.2 %)	129 (83.8 %)	1.09	0.70–1.70	0.716
Diet – Raw + Wet	No	296 (17.2 %)	1422 (82.8 %)	1 (base)		
	Yes	7 (20.6 %)	27 (79.4 %)	0.80	0.35–1.86	0.609
Diet – Complete + Wet	No	229 (17.4 %)	1087 (82.6 %)	1 (base)		
	Yes	74 (17.0 %)	362 (83.0 %)	1.03	0.77–1.37	0.837
Diet – Raw + Complete + Wet	No	289 (17.6 %)	1349 (82.4 %)	1 (base)		
	Yes	14 (12.3 %)	100 (87.7 %)	1.53	0.86–2.72	0.146
Supplement – Coconut Oil	No	275 (15.6 %)	1486 (84.4 %)	1 (base)		
	Yes	30 (18.3 %)	134 (81.7 %)	1.21	0.80–1.83	0.370
Supplement – Glucosamine	No	255 (14.3 %)	1527 (85.7 %)	1 (base)		
	Yes	50 (35.0 %)	93 (65.0 %)	3.22	2.23–4.65	<0.001
Supplement – Chondroitin	No	275 (14.9 %)	1565 (85.1 %)	1 (base)		
	Yes	30 (35.3 %)	55 (64.7 %)	3.10	1.95–4.93	<0.001
Supplement – Plaque off	No	280 (15.8 %)	1491 (84.2 %)	1 (base)		
	Yes	25 (16.2 %)	129 (84.2 %)	1.03	0.66–1.61	0.890
Supplement – Vitamin C	No	296 (15.6 %)	1600 (84.4 %)	1 (base)		
	Yes	9 (31.0 %)	20 (69.0 %)	2.43	1.10–5.39	0.029
Supplement – Multi Vitamin	No	294 (15.6 %)	1585 (84.4 %)	1 (base)		
	Yes	11 (23.9 %)	35 (76.1 %)	1.69	0.85–3.37	0.134

The multivariable model of demographic and conformational risk factors identified breed variety, age and neuter status as significantly associated with IVDD diagnosis. Of the Dachshund varieties, the MSH had 4.6 times (95 % CI 2.6–8.1), MWH 3.1 times (95 % CI 1.7–5.3) and SSH 4.5 times the odds (95 % CI 2.4–8.4) of having IVDD compared with SWH. Dogs aged between 4–6 years. 6–8 years, 8–10 years and >10 years were all at an increased odds of having IVDD compared to dogs aged 0–2 years, with dogs aged 8–10 years the group at highest odds. Entire dogs were at 0.6 times the odds (95 % CI 0.5–0.9) of IVDD compared to neutered dogs (Table 7). No interactions were identified between variables. Good final model fit was suggested by a Hosmer-Lemeshow test (*P* =0.815).

**Table 7:** Risk factors for intervertebral disc disease (IVDD) from final multivariable binary logistic regression model (OR = odds ratio, CI = confidence interval)

Variable	Category	Case	Non-Case	OR	95 % CI	*p*
Breed	SWH	18 (7.1 %)	234 (92.9 %)	1 (base)		<0.001
	MLH	46 (13.3 %)	300 (86.7 %)	1.77	0.96–3.25	0.07
	MSH	127 (17.1 %)	617 (82.9 %)	4.60	2.62–8.08	<0.001
	MWH	54 (17.7 %)	251 (82.3 %)	3.07	1.67–5.63	<0.001
	SSH	49 (24.4 %)	152 (75.6 %)	4.46	2.37–8.40	<0.001
	SLH	16 (12.6 %)	111 (87.4 %)	1.52	0.71–3.22	0.28
Age	0–2 years	1 (0.2 %)	497 (99.8 %)	1 (base)		<0.001
	2–4 years	18 (3.9 %)	443 (96.1 %)	20.13	2.67–151.66	0.004
	4–6 years	50 (13.6 %)	318 (86.4 %)	81.80	11.21–596.83	<0.001
	6–8 years	99 (33.0 %)	201 (67.0 %)	259.43	35.80–1880.22	<0.001
	8–10 years	75 (41.9 %)	104 (58.1 %)	425.29	58.04–3116.37	<0.001
	>10 years	67 (39.6 %)	102 (60.4 %)	391.21	53.23–2875.16	<0.001
Neuter status	Neutered	231 (20.9 %)	874 (79.1 %)	1 (base)		
	Entire	79 (9.1 %)	791 (90.9 %)	0.63	0.45–0.85	0.003

## Discussion

This study used as large sample of data from Dachshund owners to estimate the prevalence of IVDD, and identify lifestyle risk factors for IVDD in this breed. This study reported the prevalence of IVDD in Dachshunds to be 15.7 %, with the most commonly affected Dachshund variety the SSH (24.4 %), which were at 4.2 increased odds of IVDD compared to the SWH. These results demonstrate the high prevalence of IVDD in the Dachshund breeds when compared to previous reports of IVDD prevalence in other breeds [2, 3], and has also identified a substantial disease burden for IVDD in the SSH MSH and MWH varieties. With over 17 % difference in prevalence between the most and least affected varieties, further investigation of the genetic, conformational and lifestyle differences between these varieties is warranted to identify disease-related genes. IVDD risk is likely multifactorial and in addition to breed variety as a risk factor, a number of lifestyle based risk factors were found to be associated with IVDD, which may offer opportunities for owners and breeders to reduce the risk of IVDD in their dogs, and will be described in detail later in the discussion.

This study identified a range of factor associated with IVDD diagnosis; however, as this was a cross-sectional study, the associations identified between risk factors and IVDD may represent cases of reverse causality, in this case, where the presence of the disease leads to the presence of the risk factor. For example, owners of dogs diagnosed with IVDD may have started to supplement their dog with glucosamine and chondroitin after the event, thus associating IVDD with these supplements. Without temporal data of when owners commenced each of the factors investigated here i.e., before or after an IVDD diagnosis, the temporality and thus causality of events cannot be accurately attributed. Future studies should employ a longitudinal cohort design, following dogs over time (for example, the Non-Case group in this study), identifying lifestyle risk factors before an IVDD event occurs, and then identifying statistical associations, to overcome this limitation. We cannot make firm recommendations on IVDD risk reduction based on the results presented here; however, the factors identified as being associated with IVDD are hypothesis-generating and can be further studied in this manner to provide stronger evidence as to whether they influence IVDD risk. As such, we did not carry out multivariable models of exercise, activity and diet related variables so that all factors found to be significant at the univariable level can be further investigated.

This study used owner-reported data, from a broad range of owners and breeders of Dachshunds, and included IVDD treated at both first opinion and referral level in an effort to prevent a bias towards only the most severe cases. This is important as only including the most severe cases may have led to underestimates of prevalence, and is a common limitation of referral studies [25]. Our study population was self-selected and biased towards dogs that owners were prepared to submit details for, and thus it is possible that owners of dogs with IVDD did not submit details of their dog leading to underestimates in prevalence; however, the opposite may also apply. This study included all veterinarian-diagnosed episodes of IVDD, from spinal pain to paralysis. As not all cases underwent advanced diagnostic imaging it is possible that some cases were falsely diagnosed with IVDD (thus overestimating the prevalence). Finally, this study reported prevalence, not incident cases, and therefore milder cases that are more likely to live longer were more likely to be present in such a study, thus distorting prevalence estimates.

The prevalence of IVDD reported in this study is lower than in previous reports, for example in a study utilising insurance data, 24.4 % of Miniature Dachshunds were affected, where only 16.3 % were in our study [26]. The slightly prevalence seen here may be due to a variety of reasons including geographical differences, which may lead to genetic divergence between the populations that may affect IVDD risk, and the age of dogs included in the study. In addition, in our study, dogs that only received a presumptive diagnosis from their owner were excluded from analyses due to the inherent uncertainty of their IVDD status. With further diagnostic work-up it is possible that some of these dogs may have been classed as Cases, thus increasing the prevalence estimate.

Older Dachshunds had a higher risk of IVDD than younger Dachshunds, which is unsurprising as both disc extrusions and protrusions have degenerative aetiologies and are therefore more likely to be found in older animals, with this effect previously seen [9, 10]. Disc extrusions commonly occur between 3–7 years of age [2], and by 6–7 years of age, between 50 and 68.7 % of all discs have undergone fibrous changes that may precede IVDD [5]. As such, it is possible that some of the unaffected dogs in this study may go on to become affected in the future. Previously reported associations with conformation were not found in this population [10, 17]. It is possible that owner-derived measurements were not sufficiently reliable, or that due to this being a Dachshund-only study, differences between individuals were not sufficiently different to detect effects.

Risk factors that can be managed by an owner during a dog’s lifetime are of particular interest to existing pet owners, to attempt to avoid episodes of IVDD in their dog. Previous studies have already identified lifestyle-based risk factors including duration of exercise and moderate stair climbing, that can reduce the risk of disc disease [19]. This study has identified several more exercise and activity-related factors that were associated with IVDD risk. Dogs that exercised for less than 30 min per day were found to be at an increased odds of having IVDD, while dogs that exercised for more than 1 h per day were at a decreased odds of having IVDD. In parallel with this finding, dogs that were considered to be highly or moderately active by their owners were at a decreased odds of IVDD compared to dogs considered not at all active. It is possible that this is a case of reverse causality, and that owners of dogs that experienced an episode of IVDD were subsequently exercised less, thus associating exercise levels and IVDD. Alternatively, this result may reflect that dogs that receive a higher level of exercise have a correspondingly increased level of musculature that supports the spine and reduces the risk of disc protrusion or herniation. It is also possible that increased duration of exercise leads to improved nutrition of the disc and lowers the likelihood of initial mineralisation, with nutrient diffusion believed to be facilitated by moderate intermittent hydrostatic pressure, e.g., during physical exercise. Obesity has previously been identified as a risk factor for IVDD in a variety of dog breeds [10]. Body condition score was not found to be a risk factor for IVDD in this study, or statistically associated with level of exercise, and thus the effect of exercise found here does not indicate that dogs receiving less exercise were more overweight, leading to an elevated risk of IVDD. The fact that previous reports of BCS as a risk factor for IVDD could not be replicated here could indicate that owners were unreliable in their reports of BCS, or that the 5-point BCS system used here was not sufficiently sensitive to differentiate between dogs, compared with the 9-point scale previously used [10, 27]. Future studies that objectively compare the body composition (e.g., % fat, muscle thickness) between dogs with and without IVDD may provide further insights into physical risk factors for IVDD.

A previous study identified moderate, rather than infrequent, stair climbing was associated with reduced IVDD risk in SWH Dachshunds [19], the variety at lowest risk in our study. The current study also found a trend towards dogs that did not use stairs, or only one step in/out of the house each day being at an increased risk of IVDD compared to those that used a flight of stairs daily, although this effect was non-significant at *P* < 0.001 level. Finally, the current study found that dogs that were not allowed to jump on and off furniture were at an increased odds of IVDD compared to those who were allowed. This activity is anecdotally discouraged by vets and breeders of Dachshunds due to fears that it may trigger a disc extrusion [28]. It is plausible that excessive or prolonged load on the intervertebral joints may decrease the integrity of the disc, leading to an extrusion. As such, this result may be a case of reverse causality, where dogs who have experienced an IVDD episode are no longer allowed to jump on and off furniture. However, it is possible that dogs who regularly jump on and off furniture may develop more adequate musculature that supports the spine compared to those who do not, and thus this may be a protective effect against IVDD.

Two novel lifestyle variables were associated with a reduced risk of IVDD: being involved in conformational showing at either Championship shows or Open shows. There are several potential explanations for this novel finding, for example, dogs that compete in these shows may have IVDD-related genetic differences to those who do not and thus be at lower risk, their owners of show dogs may be more aware of IVDD and thus take measures to reduce their dog’s risk, or their owners may be less likely to report incidences of IVDD for commercial reasons. It is also possible that dogs who experience an episode of IVDD that were previously shown are no longer used in showing, and thus the population involved in Championship or Open shows are biased towards those who have not yet experienced an IVDD episode. The positive association identified between neutering and IVDD may also be similarly explained, with some dogs experiencing IVDD neutered after this event to avoid using these dogs in breeding (*n* = 30), and thus introducing reverse causality.. It is also possible that there are unidentified hormonal influences upon IVDD risk, indeed, of the IVDD affected dogs that had been neutered (*n* = 229), the majority (86.9 %, *n* = 199) had been neutered before their first IVDD event. Longitudinal studies would again be beneficial to clarify these temporal relationships. Examination of the pedigree information, and potential development of estimated breeding values (EBVs) for IVDD, could determine genetic risk more effectively [29].

Diet has not previously been studied in relation to IVDD risk. No effects were identified in the current study between the dog’s main diet or provision of treats and IVDD. Two associations with supplements were identified, with dogs supplemented with glucosamine or chondroitin at an increased odds of IVDD than those that were not. These supplements are provided by owners as preventative measures for IVDD; however, to the author’s knowledge, their effect on disc degeneration has not been clinically reported, and only weak evidence exists to support a positive effect of glucosamine/chondroitin sulfate in dogs for the treatment of osteoarthritis [30].

The investigation of lifestyle variables as potential risk factors for disease is a promising area of study in veterinary science, and is commonly used in human medical science. In humans, lifestyle risk factors for lumbar disc disease have been investigated, and in a systematic review, factors including smoking, high serum cholesterol levels and atheromatous lesions in the aorta were associated with disc degeneration and lower back pain [31]. Promoting lifestyles conducive to good health is essential to fulfilling the needs of animals, and studies that provide an evidence-base for a healthy canine lifestyle are needed to objectively advise owners.

## Conclusion

This study reports that IVDD is commonly diagnosed in all six varieties of Dachshund, with three varieties at higher risk. The high prevalence to IVDD in these varieties necessitates the development and promotion of effective plans to reduce risk via improved breeding practices, and lifestyles that avoid risk factors associated with IVDD. Several lifestyle risk factors were identified in this study that were associated with IVDD risk. These results can inform an evidence-based approach to advising Dachshund owners as to which activities to avoid or promote in their dogs to mitigate the risk of IVDD, advise breeders regarding the breed-associated risks of IVDD, and are hypothesis-generating for future prospective studies of IVDD risk.

## Additional files

Additional file 1: Table S1. Popularity of specific activities participated in by Dachshunds (n=2031). **Table S2.** Prevalence of diet, treats and supplements fed to Dachshunds (*n*=2031). **Table S3.** Prevalence of diagnoses other than IVDD in a population of Dachshunds (*n*=2013) (CI= confidence interval). (DOCX 14 kb)
Additional file 2: DachsLife Data. (XLSX 1280 kb)

## Abbreviations

BLHW: Back length: height at the wither
CT: Computed tomography
IQR: Inter-quartile range
IVDD: Intervertebral disc disease
IVDE: Intervertebral disc extrusion
MLH: Miniature long-haired
MRI: Magnetic resonance imaging
MSH: Miniature smooth-haired
MWH: Miniature wire-haired
QOL: Quality of life
SLH: Standard long-haired
SSH: Standard smooth-haired
SWH: Standard wire-haired
TLHW: Total length: height at the withers
