# Supplementary material for: DachsLife 2015: an investigation of lifestyle associations with the risk of intervertebral disc disease in Dachshunds
Source: Canine Genet Epidemiol. 2016 Nov 5;3:8. doi: 10.1186/s40575-016-0039-8 (PMC5097381; doi:10.1186/s40575-016-0039-8)
Supplement: Additional file 1: Table S1. — Popularity of specific activities participated in by Dachshunds (n=2031). Table S2. Prevalence of diet, treats and supplements fed to Dachshunds (n=2031). Table S3. Prevalence of diagnoses other than IVDD in a population of Dachshunds (n=2013) (CI= confidence interval). (DOCX 14 kb) [file 40575_2016_39_MOESM1_ESM.docx]

Additional files

Table S1. Popularity of specific activities participated in by Dachshunds (n=2031)

| **Activity** | **Yes % (n)** |
| --- | --- |
| Obedience | 21.4% (427) |
| Participation in group walks | 15.9% (317) |
| Showing at fun/charity/exemption shows | 12.1% (242) |
| Showing at Kennel Club Championship shows | 11.4% (228) |
| Showing at Kennel Club Open shows | 11.1% (221) |
| Canine Good Citizen | 8.7% (173) |
| Mini agility | 4.2% (83) |
| Earth/Working trials | 2.8% (56) |
| Therapy dog (Pets as Therapy) | 2.0% (39) |
| Heelwork to music | 0.4% (7) |
| Cani-X | 0.3% (5) |

Table S2. Prevalence of diet, treats and supplements fed to Dachshunds (n=2031)

| **Category** | **Sub-category** | **% of all dogs** |
| --- | --- | --- |
| Diet | Complete dry | 40.6% (732) |
|  | Mix of complete and wet | 24.8% (447) |
|  | Wet | 9.2% (165) |
|  | Mix of raw and complete | 9.1% (164) |
|  | Raw (BARF) | 7.8% (141) |
|  | Mix of raw / complete / wet | 6.5% (118) |
|  | Mix of raw and wet | 1.9% (35) |
| Treats | Dog treats | 53.8% (1093) |
|  | Dog biscuits | 37.8% (768) |
|  | Other treats | 22.8% (463) |
|  | Table scraps | 19.7% (401) |
|  | Other human food (e.g. scrambled egg) | 16.6% (337) |
| Supplements | Vitamin C | 1.6% (31) |
|  | Multivitamins | 2.4% (47) |
|  | Chondroitin | 4.4% (87) |
|  | Glucosamine | 7.3% (145) |
|  | Plaque-off | 8.0% (159) |
|  | Coconut oil | 8.5% (168) |
|  | Other | 19.9% (393) |

Table S3. Prevalence of diagnoses other than IVDD in a population of Dachshunds (n=2013) (CI= confidence interval)

| **Disorder** | **Cases** | **% Prevalence** | **95% CI** |
| --- | --- | --- | --- |
| Adverse reaction to Vaccination | 61 | 3.0 | 2.26-3.74 |
| Arthritis | 63 | 3.1 | 2.35-3.85 |
| Auto-immune Disease | 18 | 0.9 | 0.49-1.31 |
| Blindness ⃰ | 27 | 1.3 | 0.81-1.79 |
| Cancers or Tumours (except Mammary) | 27 | 1.3 | 0.81-1.79 |
| Cushing's | 11 | 0.5 | 0.19-0.81 |
| Deafness | 19 | 0.9 | 0.49-1.31 |
| Distichiasis | 8 | 0.4 | 0.13-0.67 |
| Epilepsy | 24 | 1.2 | 0.73-1.67 |
| Heart Disease | 11 | 0.5 | 0.19-0.81 |
| Heart Murmur | 72 | 3.5 | 2.7-4.3 |
| Kidney Disease | 1 | 0.0005 | 0.00-0.01 |
| Lafora Disease ⃰ | 6 | 0.3 | 0.06-0.54 |
| Liver Disease | 10 | 0.5 | 0.19-0.81 |
| Mammary Tumour(s) | 23 | 1.1 | 0.65-1.55 |
| Patella Luxation | 28 | 1.4 | 0.89-1.91 |
| Skin Allergy | 266 | 13.1 | 11.63-14.6 |

⃰ clinical signs, not DNA test
